# Exploring Evolution to Uncover Insights Into Protein Mutational Stability

**DOI:** 10.1093/molbev/msae267

**Published:** 2024-12-30

**Authors:** Pauline Hermans, Matsvei Tsishyn, Martin Schwersensky, Marianne Rooman, Fabrizio Pucci

**Affiliations:** Computational Biology and Bioinformatics, Université Libre de Bruxelles, Brussels 1050, Belgium; Interuniversity Institute of Bioinformatics in Brussels, Brussels 1050, Belgium; Computational Biology and Bioinformatics, Université Libre de Bruxelles, Brussels 1050, Belgium; Interuniversity Institute of Bioinformatics in Brussels, Brussels 1050, Belgium; Computational Biology and Bioinformatics, Université Libre de Bruxelles, Brussels 1050, Belgium; Interuniversity Institute of Bioinformatics in Brussels, Brussels 1050, Belgium; Computational Biology and Bioinformatics, Université Libre de Bruxelles, Brussels 1050, Belgium; Interuniversity Institute of Bioinformatics in Brussels, Brussels 1050, Belgium; Computational Biology and Bioinformatics, Université Libre de Bruxelles, Brussels 1050, Belgium; Interuniversity Institute of Bioinformatics in Brussels, Brussels 1050, Belgium

**Keywords:** protein evolution, protein mutational landscape, coevolution and epistasis, protein thermodynamic stability

## Abstract

Determining the impact of mutations on the thermodynamic stability of proteins is essential for a wide range of applications such as rational protein design and genetic variant interpretation. Since protein stability is a major driver of evolution, evolutionary data are often used to guide stability predictions. Many state-of-the-art stability predictors extract evolutionary information from multiple sequence alignments of proteins homologous to a query protein, and leverage it to predict the effects of mutations on protein stability. To evaluate the power and the limitations of such methods, we used the massive amount of stability data recently obtained by deep mutational scanning to study how best to construct multiple sequence alignments and optimally extract evolutionary information from them. We tested different evolutionary models and found that, unexpectedly, independent-site models achieve similar accuracy to more complex epistatic models. A detailed analysis of the latter models suggests that their inference often results in noisy couplings, which do not appear to add predictive power over the independent-site contribution, at least in the context of stability prediction. Interestingly, by combining any of the evolutionary features with a simple structural feature, the relative solvent accessibility of the mutated residue, we achieved similar prediction accuracy to supervised, machine learning-based, protein stability change predictors. Our results provide new insights into the relationship between protein evolution and stability, and show how evolutionary information can be exploited to improve the performance of mutational stability prediction.

## Introduction

Understanding how mutations impact protein thermodynamic stability is of fundamental interest for a wide range of applications spanning from protein design (Coluzza 2017; Pucci et al. 2022) to the interpretation of genetic variants (Gerasimavicius et al. 2020; Iqbal et al. 2020). Evolution has largely been used to guide computational or experimental mutagenesis aimed at improving protein stability, the latter being the primary driver of evolution (Bloom et al. 2006; Zeldovich et al. 2007; Kurahashi et al. 2018).

Directed evolution methods, which aim to replicate evolutionary processes in vitro through iterative steps of random mutagenesis, selection, and amplification, have played a fundamental role in the (re)design of new proteins with increased stability (Packer and Liu 2015; Arnold 2018; Wang et al. 2021). Computational mutagenesis approaches have been extensively improved in the last decade (Dehouck et al. 2011; Pires et al. 2014; Montanucci et al. 2019; Sanavia et al. 2020; Pucci et al. 2022) and have often been combined with experimental methods to limit the time-consuming exploration of the vast protein sequence space (Coluzza 2017; Korendovych 2018). Recently, deep learning techniques have also been introduced to the field, but their application has often been constrained by the limited availability of experimental thermodynamic data. New high-throughput stability assays, such as the assay recently developed in Tsuboyama et al. (2023), are currently changing the field by generating folding stability measurements on an impressively large scale, providing essential information to better understand protein stability and to constitute sufficiently large datasets to train deep learning models.

Some current state-of-the-art models for stability prediction (Fariselli et al. 2015; Chen et al. 2020b; Høie et al. 2022) already take advantage of evolution and of the huge amount of sequence data available in metagenomic databases (Hou et al. 2022). They extract evolutionary information from multiple sequence alignments (MSAs) of proteins homologous to a query protein, which is used to predict how mutations affect protein stability. Although evolution and protein stability are known to be closely related (Ota et al. 2018), they are not always directly linked. Indeed, functional regions such as catalytic or binding sites are not at all optimized for stability, whereas they clearly show a very strong conservation (Chen et al. 2020a; Hou et al. 2023). In addition, several open questions are still being debated in the field. For example, it remains unclear whether the effects of mutations are conserved throughout evolution (Ashenberg et al. 2013; Starr and Thornton 2016). This question is related to the intriguing role of epistatic effects (Starr and Thornton 2016) in shaping protein evolution and stability, which is still not fully understood. While some evidence points to their essential role (Pollock et al. 2012; Pollock and Goldstein 2014; Goldstein and Pollock 2017), other studies suggest otherwise (Ashenberg et al. 2013). From a computational perspective, the inclusion of epistatic contributions in evolutionary models generally seems to better capture the protein mutational fitness landscape (Figliuzzi et al. 2016; Hopf et al. 2017), though this is not always the case and often depends on the specific protein considered (Laine et al. 2019).

Finally, from a practical point of view, it is unclear what is the best way to generate and manage MSAs in order to extract evolutionary signals that can improve protein stability prediction and protein design. In the last couple of years, much attention has been paid to optimizing or subsampling the input MSAs to improve downstream tasks such as predicting the three-dimensional (3D) structures of proteins and of their complexes (Ovchinnikov et al. 2017; Jumper et al. 2021; Mirdita et al. 2022; Petti et al. 2023; Wallner 2023). A recent analysis on how MSA construction impacts the outcome of mutational fitness prediction (Abakarova et al. 2023) presented interesting findings, in particular that prediction accuracy does not always correlate with alignment depth; evolutionary information extracted from shallow MSAs can sometimes predict the effect of mutations on protein fitness with high accuracy. Adding homologous sequences that are evolutionary distant from the query sequence does thus not necessarily improve predictions (Pucci et al. 2020; Abakarova et al. 2023).

We leveraged the massive amount of protein stability data generated in Tsuboyama et al. (2023) and analyzed how to optimally extract evolutionary information from MSAs for mutational stability predictions. One of our goals being to gain insight into the relationship between protein stability and evolution, we compared the ability of various kinds of evolutionary models to predict protein stability changes upon mutations. We also highlighted the relationships between residue solvent accessibility (RSA) which is a simple structural feature, the evolutionary conservation of these residues, and the effect of their substitutions on protein stability. Based on these relationships, we devised a way to modulate evolutionary features with RSA, resulting in simple unsupervised models that yield results similar to those of more complex state-of-the-art protein stability change predictors.

## Methods

### MSA Construction

For each query sequence, we generated MSAs using the iterative JackHMMER method (Finn et al. 2011) against a given sequence dataset. Basically, JackHMMER performs a quick sequence similarity search on the considered sequence dataset to build a first MSA, which is used to set up a hidden Markov model (HMM). This HMM is then searched against the sequence dataset to select new sequences and construct the next MSA and HMM, and so on. Each iteration refines both the MSA and the HMM used for its construction.

In order to assess the relevance of the MSAs for stability change predictions and compare different ways of constructing them, we tested various numbers of iterations and E-value thresholds in JackHMMER (see Section Impact of MSA construction and curation). We also evaluated different sequence datasets. We primarily used the UniRef90 set (Suzek et al. 2015), but also tested searching in two larger datasets: UniRef100 and Metagenomics, a metagenomic dataset constructed by merging UniRef90 (Suzek et al. 2015), the Big Fantastic Database (Steinegger et al. 2019), the Gut Phage Database (Camarillo-Guerrero et al. 2021), MetaEuk (Levy Karin et al. 2020), the Metagenomic Gut Virus (MGV) (Nayfach et al. 2021), SMAG (Ma et al. 2023), TOPAZ (Alexander et al. 2023), and MGnify (Mitchell et al. 2020).

As MSAs can contain clusters of very closely related proteins, measuring the amount of information contained in them by $N_{\mathrm{tot}}$, the total number of sequences it contains, can sometimes be inaccurate. For that reason, we rather used $N_{\mathrm{eff}}$, the effective number of independent sequences, defined as the sum of the weights $w_{j}$ assigned to each sequence $s_{j}$ in the MSA (Morcos et al. 2011):


(1) $$N_{\mathrm{eff}}=\sum_{\;j=1}^{N_{\mathrm{tot}}}w_{j}$$

in order to assess the impact of the “depth” of an MSA on predictions. The weight $w_{j}$ of a sequence $s_{j}$ is defined as the inverse of $m_{j}$, the number of sequences in the MSA sharing at least 80% identity with $s_{j}$:


(2) $$w_{j}=\frac{1}{m_{j}}=\frac{1}{\left|\left\{s\in S\;|\;\text{seqid}(s_{j},s)\geq 80\%\right\}\right|}$$

where *S* is the set of sequences in the MSA. In this way, sequences without similar sequences in the dataset have a weight of one, and sequences with similar sequences are down-weighted. These weights were computed as in Morcos et al. (2011) using the **plmc** software from the EVcouplings suite (Hopf et al. 2017).

### Variant Dataset Construction

We used the recent deep mutational scanning dataset constructed in Tsuboyama et al. (2023) (accessed in February 2023), which contains about one million experimental estimations of protein folding free energy (ΔG) obtained through cDNA display proteolysis applied to 396 “wild-type” sequences. We first curated this dataset by averaging duplicate measured ΔG values for specific sequences (both “wild-type” and “mutant”). We focused on single amino acid substitutions and computed the corresponding changes in folding free energy upon mutations, defined as $\Delta \Delta G=\Delta G_{mt}-\Delta G_{wt}$, where *wt* and *mt* stand for “wild-type” and for “mutant” respectively.

To reduce protein-specific imbalances in the dataset, we employed the clustering method of CD-HIT (Fu et al. 2012) with default parameters (sequence identity threshold of 0.9). In this way, we removed closely related sequences and limited the number of “wild-type” sequences to 308. As many of these proteins have a very small number of known homologs (among which de novo designed proteins), we further refined the dataset by removing proteins whose MSA (obtained by JackHMMER on UniRef90 with one iteration and default parameters) contains less than 100 sequences. The resulting dataset, called D, contains 135,056  ΔΔG values of single-site mutations in 129 protein domains with lengths ranging from 32 to 72 amino acids. The obtained distribution of the experimental ΔΔG values is presented in supplementary section S1, Supplementary Material online. As expected, most of the mutations are destabilizing, with an average destabilizing ΔΔG of 0.82 kcal/mol.

In order to investigate the effect of MSA depth on different evolutionary models, we used the $N_{\mathrm{eff}}$ value (Eq. 1) as a criterion to partition the dataset D into subsets: $D_{10}$ ($10\leq N_{\mathrm{eff}}<100$, 17 proteins), $D_{100}$ ($100\leq N_{\mathrm{eff}}<1,000$, 27 proteins), $D_{1,000}$ ($1,000\leq N_{\mathrm{eff}}<10,000$, 41 proteins), and $D_{10,000}$, ($10,000\leq N_{\mathrm{eff}}$, 44 proteins). Values of $N_{\mathrm{eff}}$ were derived on MSAs constructed on UniRef90 with two JackHMMER iterations and an E-value threshold of $10^{-7}$, as we showed in Section ‘Impact of MSA construction and curation’ that these parameter values are optimal for stability predictions. The structures of all “wild-type” proteins were modeled using AlphaFold2 (Jumper et al. 2021).

To analyze the generalizability of our results to larger proteins, we additionally used the recently published literature-based, nonsystematic, dataset S4038 (Zheng et al. 2024). We constructed dataset L by selecting the seven proteins in S4038 which are larger than the proteins from D and for which experimental ΔΔG values of at least 60 mutations have been reported (see supplementary section S2, Supplementary Material online for details).

The complete datasets as well as the PDB structures used in this paper are available in our GitHub repository https://github.com/3BioCompBio/EvoStability.

### Residue and Mutation Properties

We first defined three evolution-based features derived from positional amino acid frequencies in an MSA. To avoid divergent values and manage the lack of information in small MSAs, we defined a way to regularize the observed amino acid frequencies. Considering the positional frequency $f_{i}(a)$ of amino acid *a* at position *i* in the MSA and the frequency f(a) of amino acid *a* in the full MSA, we defined the regularized frequencies ${\bar{\;f}}_{i}(a)$ and $\bar{\;f}(a)$ as:


(3) $$\begin{array}{rl} & {\bar{\;f}}_{i}(a)=f_{i}(a)(1-\theta )+\frac{\theta }{21}\;\text{and}\\ & \bar{\;f}(a)=f(a)(1-\theta )+\frac{\theta }{21}, \end{array}$$

where *θ* is a regularization factor that we set to 0.01, and 21 stands for the number of possible states (20 standard amino acids, and 1 gap). Using these regularized frequencies, the three evolution-based features are:

- **Conservation Index (CI)** (Calabrese et al. 2009). It is a measure of the conservation of residues at each position in an MSA:
  (4) $$\text{CI}(i)={\left[\frac{1}{2}\sum_{a\in A}{\left({\bar{\;f}}_{i}(a)-\bar{\;f}(a)\right)}^{2}\right]}^{1/2},$$
  where *A* is the set of 20 amino acids.
- **Log-Odd Ratio (LOR)** (Raimondi et al. 2016). By comparing the frequencies of wild-type (*wt*) and mutant (*mt*) amino acids in the MSA at a given position, we can statistically estimate their prevalence in their evolutionary context. The log-odd ratio of observing the *wt* amino acid with respect to the *mt* amino acid at position *i* is given by:
  (5) $$\text{LOR}(i,wt,mt)=\log\;\frac{{\bar{\;f}}_{i}(wt)}{1-{\bar{\;f}}_{i}(wt)}-\log\;\frac{{\bar{\;f}}_{i}(mt)}{1-{\bar{\;f}}_{i}(mt)}.$$
  The sign of LOR is set such that a mutation from a highly represented amino acid *wt* to a poorly represented amino acid *mt* has a positive LOR, which is comparable to a destabilizing mutation with ΔΔG>0.
- **Weighted Log-Odd Ratio (${\text{LOR}}_{w}$)**. To reduce the impact of clusters of closely related sequences in the MSA, we slightly modified the definition of LOR by rectifying the count for amino acid frequencies using the weights assigned to each sequence in the MSA (computed with **plmc** as for $N_{\mathrm{eff}}$, see Section Multiple Sequence Alignment Construction). The weighted log-odd ratio is defined as:
  (6) $$\begin{array}{rl} {\text{LOR}}_{w}(i,wt,mt)\;= & \log\;\frac{{\bar{\;f}}_{i}^{w}(wt)}{1-{\bar{\;f}}_{i}^{w}(wt)}\\ & -\log\;\frac{{\bar{\;f}}_{i}^{w}(mt)}{1-{\bar{\;f}}_{i}^{w}(mt)}, \end{array}$$
  where the weighted frequency $f_{i}^{w}(a)$ of an amino acid *a* at position *i* is defined as:
  (7) $$f_{i}^{w}(a)=\frac{\sum_{\;j}w_{j}\delta (a,a_{ij})}{\sum_{\;j}w_{j}},$$
  with $w_{j}$ is the weight of sequence *j*, $a_{ij}$ the amino acid at position *i* in sequence *j* and δ(A,B) is the Kronecker symbol, which equals one if the *A* and *B* coincide, and zero otherwise. Note that in this case, the regularization step of Eq. 3 is applied to the weighted frequencies.

We also used four mutational coevolutionary methods:

- **pycofitness** (Pucci et al. 2024) is a direct coupling analysis (DCA)-based model. DCA provide a statistical representation of a family of homologous protein sequences from a given MSA. Let $S=(a_{1},a_{2},\ldots ,a_{L})$ be a protein sequence of length *L*, where $a_{i}$ is the amino acid type at position *i*. The sequence *S* is assumed to be sampled across evolution with a probability given by the Boltzmann distribution, P(S), which can be expressed as:
  (8) $$P(S)=\frac{1}{Z}\exp\;(\;-\beta \Phi (S)),$$
  where *β* is the inverse temperature, *Z* denotes the partition function, and Φ(S) is the energy of the system:
  (9) $$\Phi (S)=-\sum_{1\leq i<j\leq L}J_{ij}(a_{i},a_{j})-\sum_{i=1}^{L}h_{i}(a_{i}),$$
  with $h_{i}(a_{i})$ and $J_{ij}(a_{i},a_{j})$ corresponding to single-site fields and coupling parameters, respectively. To infer them, pycofitness uses a pseudo-likelihood maximization approach (Ekeberg et al. 2013) (see supplementary section S6, Supplementary Material online for details). Once the parameters are inferred, the effect of mutation of residue *a* into *b* at position *i* in the sequence, ΔX(i,a,b), is computed as:
  (10) $$\begin{array}{rl} \Delta X(i,a,b) & =\Phi (a_{1},\ldots ,a_{i-1},b,a_{i+1},\ldots ,a_{L})\\ & \;-\Phi (a_{1},\ldots ,a_{i-1},a,a_{i+1},\ldots ,a_{L})\\ & =\left[h_{i}(b)-h_{i}(a)\right]+\sum_{\;j=1}^{L}\left[J_{ij}(b,a_{j})-J_{ij}(a,a_{j})\right]. \end{array}$$
- **EVcouplings** (Hopf et al. 2017, 2019) suite contains two models for predicting the effects of mutations, the epistatic and the independent-site models, which are referred to as EVcouplings-epi and EVcouplings-ind, respectively. The EVcouplings-epi model accounts for epistatis by modeling interactions between all pairs of residues using a DCA model, with coefficients inferred from the input MSA through a pseudo-likelihood maximization approach (Ekeberg et al. 2013). This method is analogous to pycofitness. EVcouplings-ind is a site-wise maximum entropy model, which does not take into account interactions between sites.
- **ArDCA** (Trinquier et al. 2021) is an epistatic method that uses an autoregressive model to compute the full probability P(S) of observing the sequence $S=(a_{1},\ldots ,a_{L})$ across evolution as the product of conditional probabilities $P(S)=P(a_{1})P(a_{2}\;|\;a_{1})\ldots P(a_{L}\;|\;a_{L-1},\ldots ,a_{1})$. The model parameters are inferred through a maximum-likelihood approach.
- **GEMME** (Laine et al. 2019) combines an epistatic and an independent-site version, which will be considered independently here. The epistatic model, referred to as GEMME-epi, infers the epistatic contributions from the MSA based on the minimal evolutionary distance between a sequence carrying the mutations and the query sequence in the evolutionary tree. The independent-site model, GEMME-ind, is based on the relative per-site frequencies of the wild-type and mutant residues.

Finally, we used one feature based on the protein 3D structure, and defined a way of combining it with the evolutionary features described above:

- **Relative Solvent Accessibility (RSA)**. The RSA of a residue at position *i*, RSA(i), is defined as the ratio (in %) of its solvent-accessible surface area in its 3D structure and in a Gly-X-Gly tripeptide in extended conformation (Rose et al. 1985). We calculated it using our in-house software MuSiC (Dalkas et al. 2014), which uses an extension of the DSSP algorithm (Kabsch and Sander 1983) and is available on the www.dezyme.com website.
- **
RSA and evolution**. As mutations in the protein core tend to be more destabilizing than mutations at the protein surface (see Section Residue solvent accessibility and protein stability), RSA is a crucial feature in estimating protein stability. For this reason, we devised a simple way of combining any mutational evolutionary score with the RSA as:
  (11) $$\begin{array}{rl} \text{RSA}⊙E(i,wt,mt)\;= & \left(\frac{100\%-\text{RSA}(i)}{100\%}\right)\\ & \cdot E(i,wt,mt), \end{array}$$
  where E(i,wt,mt) is the evolutionary score of the substitution from residue *wt* to residue *mt* at position *i*.

## Results

### Impact of MSA Construction and Curation

We started by evaluating the impact of MSA construction and curation on the ability of independent-site evolutionary scores (i.e. LOR and ${\text{LOR}}_{w}$, defined in Section Residue and Mutation Properties) to predict changes in protein stability. In particular, we tested different protein sequence datasets, numbers of JackHMMER iterations, E-value thresholds and MSA curation criteria. As evaluation metric, we computed the average per-protein Spearman correlation coefficients between the evolutionary scores and the experimental ΔΔG values from the variant dataset D. We present here the results obtained with ${\text{LOR}}_{w}$ scores, as they turn out to be slightly more predictive than LOR values.

**Fig. 1.**
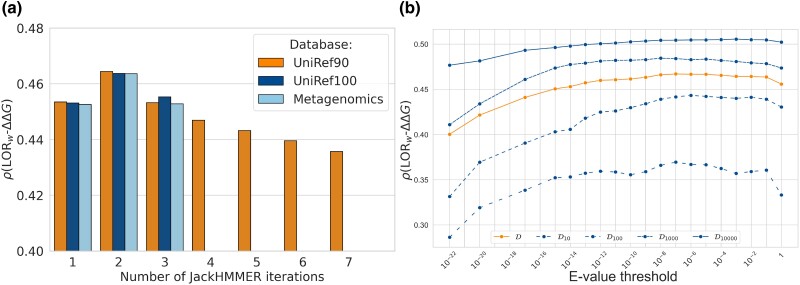
Average per-protein Spearman correlation coefficients *ρ* between ${\text{LOR}}_{w}$ and ΔΔG on dataset D as a function of: a) the protein sequence dataset and the number of JackHMMER iterations and b) the E-value thresholds between $10^{-22}$ and 1 using UniRef90 and two JackHMMER iterations (on D and its subsets $D_{10}$, $D_{100}$, $D_{1,000}$, and $D_{10,000}$).

For the number of JackHMMER iterations (ranging from 1 to 7), corresponding to successive levels of MSA refinement, we found that MSAs obtained with two iterations yield ${\text{LOR}}_{w}$ scores that correlate best with experimental ΔΔG values (Fig. 1a). This is true regardless of the sequence dataset used. This result is statistically significant; the *P*-values for the comparison of correlations and the method to derive them are provided in supplementary section S3, Supplementary Material online.

Sequences added during the second iteration contribute to the diversity of the MSAs, which is beneficial for predicting changes in protein stability. Subsequent iterations appear to introduce noise in the MSA rather than adding any useful information.

We also constructed MSAs from three sequence datasets: UniRef90, UniRef100, and Metagenomics (described in Section Multiple Sequence Alignment Construction). As represented in Fig. 1a, the ${\text{LOR}}_{w}$-ΔΔG Spearman correlation coefficients are almost identical irrespective of the sequence dataset used. The use of larger sequence datasets enriches the MSAs with homologous sequences. However, this neither increases nor decreases the correlation, which means that the additional sequences probably duplicate information already present in the MSAs constructed with the smaller UniRef90 sequence dataset. Note that this is different from what happens in contact prediction and protein structure prediction, where querying large metagenomics datasets for the input MSA construction seems to boost method performance (Ovchinnikov et al. 2017; Jumper et al. 2021).

In addition to the factors mentioned above, the quality of an MSA also depends on the E-value threshold used to construct it. We tested E-value thresholds between 1 and $10^{-22}$, knowing that the default value in JackHMMER is $10^{-3}$. As shown in Fig. 1b, the ${\text{LOR}}_{w}$-ΔΔG correlation is relatively stable for E-values between $10^{-2}$ and $10^{-8}$. The highest correlation coefficient (0.467) is reached for an E-value threshold of $10^{-7}$. For E-value thresholds below $10^{-13}$, the correlation drops significantly, since the obtained MSAs become significantly smaller and composed of sequences that are very close to the query. Following the above analysis, in the rest of the article we will use MSAs constructed from UniRef90 with two JackHMMER iterations and an E-value threshold of $10^{-7}$.

We also evaluated the effect of MSA curation by removing sequences based on their gap ratio and sequence identity with the query sequence. We found almost no effect of the curation on the ${\text{LOR}}_{w}$-ΔΔG Spearman correlation coefficients, regardless of the sequence dataset used (see supplementary section S4, Supplementary Material online). While MSA curation has been reported as an important step for better predictions (Laine et al. 2019), its effect is marginal on dataset D. This is probably due to the fact that D only contains small protein domains, which makes MSA construction simpler. For instance, larger, multidomain proteins usually have MSAs with sequences that only partially cover the query sequence, on which curation could have a stronger effect.

**Fig. 2.**
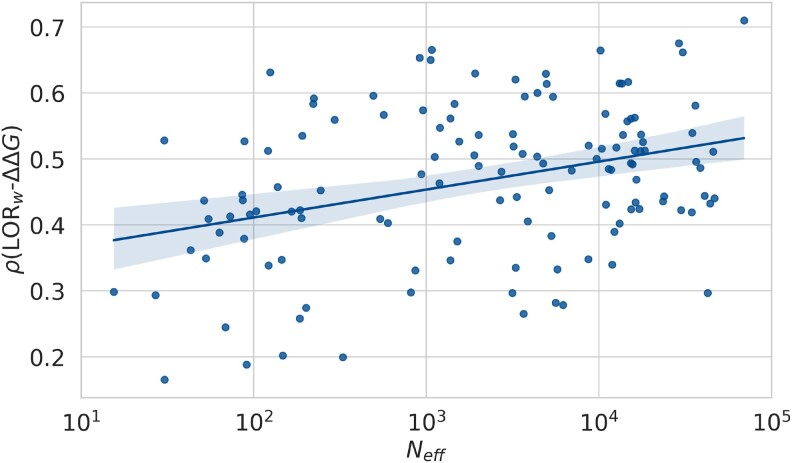
Per-protein Spearman correlation coefficients *ρ* as a function of the effective number of sequences $N_{\mathrm{eff}}$ (in $\log_{10}$ scale) in the MSAs (using UniRef90, two JackHMMER iterations and E-value threshold of $10^{-7}$).

Finally, we leveraged the large-scale and systematic nature of the deep mutational scanning dataset D to explore the impact of $N_{\mathrm{eff}}$, the effective number of sequences in an MSA, on the correlation between evolutionary scores and protein stability changes. While it is well known that the depth of an MSA increases its ability to make predictions (Hopf et al. 2017; Ovchinnikov et al. 2017; Laine et al. 2019), we are now able to more precisely quantify this relation and to further explore its application to protein stability. As shown in Fig. 2, MSAs with higher $N_{\mathrm{eff}}$ consistently lead to stronger ${\text{LOR}}_{w}$-ΔΔG correlations (and this holds true for other evolutionary models). This ensures that the MSA better represents the diversity of homologous proteins. Indeed, for the best E-value threshold ($10^{-7}$), the average Spearman correlation coefficients between ${\text{LOR}}_{w}$ and ΔΔG are equal to 0.37 on $D_{10}$, 0.44 on $D_{100}$, 0.48 on $D_{1,000}$, and 0.50 on $D_{10,000}$. However, it is worth noting that there are substantial differences in performance between proteins, even between proteins with very similar $N_{\mathrm{eff}}$ values. The wide range of correlations (from 0.2 to 0.7) can thus not be fully attributed to $N_{\mathrm{eff}}$ alone. For instance, some proteins with very shallow MSAs, composed of only a few dozen sequences, are surprisingly predicted quite accurately by evolutionary models. This phenomenon is not limited to ${\text{LOR}}_{w}$; proteins for which ΔΔG is well predicted by one evolutionary model tend to be well predicted by the other models as well, and vice versa.

### Divergent Predictions on Highly Similar Proteins

To explore the strong variability between proteins in how well evolutionary models describe their stability, we focused on two proteins from D with high sequence identity but notable differences in prediction performance. Both are engineered 57-residue β1 domains of the streptococcal G protein. The first, $G\beta 1_{\;pH}$, is a pH-sensitive mutant of *Finegoldia magna* whose sequence is identical to that of the PDB structure 2ZW1 (Watanabe et al. 2009) with the substitution V54S (called 2zw1_A_0-56_V54S in our dataset). The second, $G\beta 1_{T_{m}}$, is a redesigned hyperthermophilic heptamutant from *Streptococcus sp. G148*; its sequence is identical to that of the PDB structure 1 GB4 (Malakauskas and Mayo 1998) with the substitution F53D (referenced as 1gb4_A_ 1-57_F53D).

**Fig. 3.**
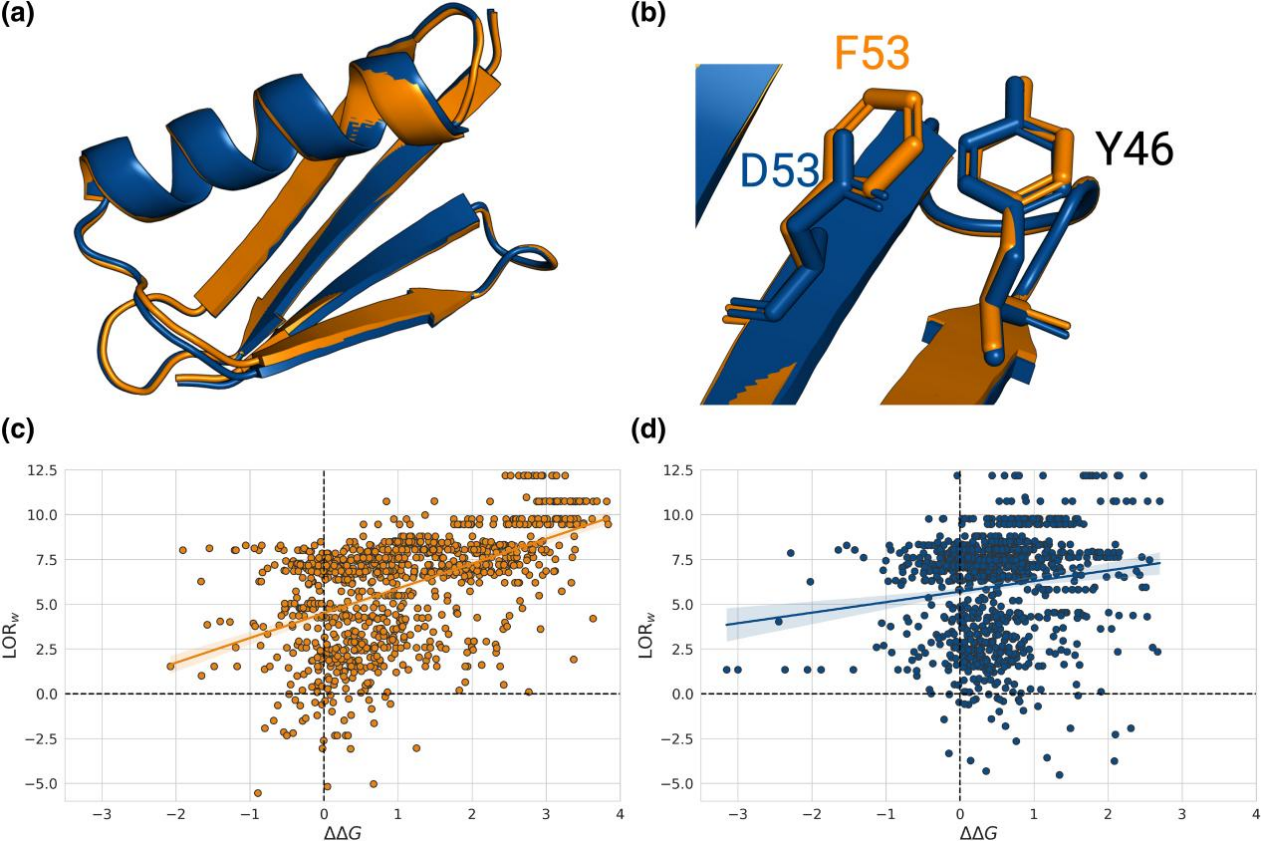
a–b) Superimposed structures of proteins $G\beta 1_{\;pH}$ (in orange) and $G\beta 1_{T_{m}}$ (in blue). b) Interactions between residues 46 and 53. c–d) Comparison between ${\text{LOR}}_{w}$ scores and experimental ΔΔG values for $G\beta 1_{\;pH}$ (c, in orange) and $G\beta 1_{T_{m}}$ (d, blue).

The two proteins share 70% sequence identity and very similar 3D structures, with a root mean square deviation (RMSD) of their C_α_ atoms of 0.4 Å (Fig. 3a). However, they show very different results in terms of ${\text{LOR}}_{w}$-ΔΔG Spearman correlation coefficients: ρ=0.53 and 0.17 for $G\beta 1_{\;pH}$ and $G\beta 1_{T_{m}}$, respectively (Table 1 and Fig. 3c–d).

**Table 1.** Spearman correlation coefficients *ρ* between experimental ΔΔG values and scores obtained with ${\text{LOR}}_{w}$, pycofitness (PCF) (Pucci et al. 2024) and PoPMuSiC (PoP) (Dehouck et al. 2011) for the two Gβ1 proteins

Protein	Length (aa)	$N_{\mathrm{eff}}$	*ρ*(${\text{LOR}}_{w}$-ΔΔG)	*ρ*(PCF-ΔΔG)	*ρ*(PoP-ΔΔG)
$G\beta 1_{\;pH}$	57	30.3	0.53	0.54	0.63
$G\beta 1_{T_{m}}$	57	30.5	0.17	0.10	0.55

Since the two proteins have a close evolutionary relationship, they have almost identical MSAs and their evolutionary scores are expected to be similar. This is indeed the case: the Spearman correlation between their ${\text{LOR}}_{w}$ values at positions sharing the same amino acid is equal to 0.99. Notably, however, their ΔΔG scores at the same positions display a much lower Spearman correlation of 0.75. This suggests that epistatic effects, which are not captured by ${\text{LOR}}_{w}$ scores, play an important role in the stability properties of these proteins.

An example of a particularly strong epistatic effect can be observed by looking at residue 46, which is occupied by a tyrosine in both $G\beta 1_{\;pH}$ and $G\beta 1_{T_{m}}$. This position is highly conserved, with 98% of tyrosine in both MSAs; it thus has a strongly unfavorable ${\text{LOR}}_{w}$ score for substitutions. However, while mutations at that position are all strongly destabilizing for $G\beta 1_{\;pH}$ (average ΔΔG of 3.0 kcal/mol), their effect is only mildly destabilizing to neutral for $G\beta 1_{T_{m}}$. Analyzing this residue in its 3D structure, we found it to be located in a partially buried region and to interact with residue 53. It forms a stabilizing π−π (parallel-displaced) interaction with F53 in $G\beta 1_{\;pH}$, and an anion−π interaction with D53 in $G\beta 1_{T_{m}}$, known to be barely stabilizing (Fig. 3b). Phenylalanine at position 53 is highly conserved in evolution, whereas aspartate at this position only occurs in the sequence of $G\beta 1_{T_{m}}$. The F53D substitution from $G\beta 1_{\;pH}$ to $G\beta 1_{T_{m}}$ has thus a strong epistatic effect on position 46: in short, it changes Y46 mutations from very destabilizing to stabilizing or neutral, an effect that cannot be captured by independent-site ${\text{LOR}}_{w}$ scores. As a consequence, ${\text{LOR}}_{w}$ is less accurate for Y46 mutations in $G\beta 1_{T_{m}}$ than in $G\beta 1_{\;pH}$.

Coevolution-based models that account for epistatic interactions should perform better and capture these effects. Unfortunately, this does not appear to be the case, as the mean pycofitness scores for all substitutions of Y46 are the same for both proteins. In addition, the Spearman correlations of ΔΔG values with pycofitness scores are very similar to those with ${\text{LOR}}_{w}$ scores for both proteins, as shown in Table 1, suggesting a difficulty in efficiently learning epistatic patterns. Similar results were observed for epistatic models other than pycofitness. We will return to the ability of such models to inform about protein stability in the next subsection.

Finally, we compared the results of evolutionary models with those of PoPMuSiC (Dehouck et al. 2011), a structure-based ΔΔG prediction model that relies on statistically derived physical features. Although a small difference in performances is still observed between $G\beta 1_{\;pH}$ and $G\beta 1_{T_{m}}$, PoPMuSiC is able to better capture the effect of mutations in both proteins, with correlation values of 0.63 and 0.55, respectively.

These observations show that the ability of evolutionary information to explain stability differs from one protein to another. Although we were able to observe and analyze such differences on a per-protein basis, we were unable to identify biophysical protein characteristics or properties of their MSAs that could consistently explain this variability.

For example, although we interpreted that the low prediction accuracy of Y46 mutations in $G\beta 1_{T_{m}}$ was due to the interacting residue at position 53 being occupied by an unusual amino acid, this cannot be generalized to the entire sequence. Rather, it appears that a few key positions, such as 53, have complex epistatic effects on the mutational landscape and make the effects of mutations less predictable using only their evolutionary history (Park et al. 2022). Analyses performed on experimental deep mutational scanning data of domain *β*1 of protein G reveal that a minority of pairs (5%) exhibit significant epistatic effects on structural stability, but that a larger fraction (30%) exhibit weaker forms of epistasis (Olson et al. 2014) that are even more difficult to detect.

### Assessing Independent-Site and Epistatic Evolutionary Models for ΔΔG Prediction

**Fig. 4.**
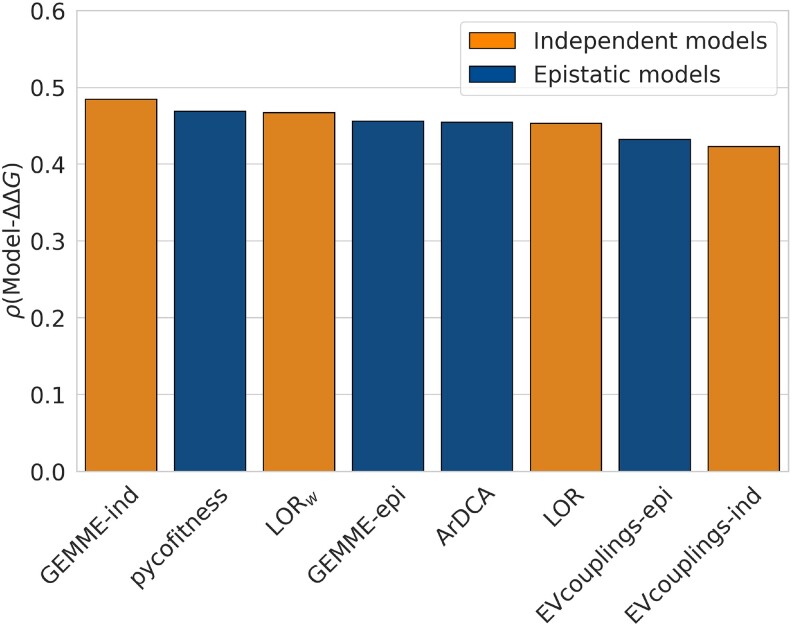
Average per-protein Spearman correlation coefficients *ρ* between experimental ΔΔG values and evolutionary scores derived from the tested independent-site models (in orange) and epistatic models (in blue), on dataset D.

We evaluated the ability of various evolution-based methods to detect the impact of mutations on protein stability. More precisely, we tested the independent-site models LOR, ${\text{LOR}}_{w}$, GEMME-ind (Laine et al. 2019), and EVcouplings-ind (Hopf et al. 2019), as well as the epistatic models pycofitness (Pucci et al. 2024), ArDCA (Trinquier et al. 2021), GEMME-epi (Laine et al. 2019), and EVcouplings-epi (Hopf et al. 2019). For that purpose, we computed the average per-protein Spearman correlation coefficient between these evolutionary scores and experimental ΔΔG values on dataset D and on its subsets (split by MSA depth, see Section Variant Dataset Construction). The performance of the evolutionary models is shown in Fig. 4 and Table 2 (*P*-values for pairwise correlation comparisons and the method to derive them are provided in supplementary section S3, Supplementary Material online).

**Table 2.** Average per-protein Spearman correlation coefficients *ρ* between experimental ΔΔG values and evolutionary scores derived from independent-site and epistatic models, on dataset D and its $N_{\mathrm{eff}}$-dependent subsets

Method	Type	D	$D_{10}$	$D_{100}$	$D_{1,000}$	$D_{10,000}$
GEMME-ind	Independent	0.48	0.38	0.44	0.51	0.53
pycofitness	Epistatic	0.47	0.38	0.43	0.49	0.50
${\text{LOR}}_{w}$	Independent	0.47	0.37	0.44	0.48	0.50
GEMME-epi	Epistatic	0.46	0.37	0.42	0.48	0.49
ArDCA	Epistatic	0.45	0.25	0.39	0.50	0.53
LOR	Independent	0.45	0.35	0.43	0.47	0.50
EVcouplings-epi	Epistatic	0.43	0.36	0.42	0.43	0.47
EVcouplings-ind	Independent	0.42	0.36	0.42	0.42	0.45

First, we observe that the performance is in general surprisingly good for evolutionary features, which are not always directly related to protein stability. Indeed, a low-frequency mutant residue in an MSA does not necessarily imply reduced protein stability, but can be caused by a different effect such as reduced function. Moreover, regardless of the method tested, the correlations are, on the average, better for proteins that have a large number of homologs, as expected. Correlation coefficients close to 0.5 are reached for all tested methods on dataset $D_{10,000}$. However, the performance remains surprisingly good, albeit lower, for shallow MSAs, with correlation coefficients in the 0.25–0.4 range on dataset $D_{10}$. Most importantly, while epistatic models are more complete and have already established their relevance in multiple applications (Cocco et al. 2018), simple independent-site methods appear to be equally predictive of stability changes (with GEMME-ind statistically significantly outperforming all epistatic models). Whether this trend is due to the relatively low impact of epistatic effects on stability or to the poor ability of these models to describe mutational effects remains to be understood.

In summary, evolutionary-based features are able to accurately estimate stability changes upon mutations. Independent-site models, which assume that residues have evolved independently of their context, tend to perform as well or even slightly better than more complex epistatic models when correlated to ΔΔG, even though the latter take into account epistatic interactions that are known to play an important role in the evolutionary trajectory of proteins (Starr and Thornton 2016; Dasmeh and Serohijos 2018; Park et al. 2022).

### Improving Performances of Epistatic Models

The surprising result of the previous section, that epistatic models, despite being more complex, do not perform better than simpler independent-site models, led us to further investigate the broader question of the role of covariation in predicting mutational effects. It is well known that in DCA methods, the inference of couplings often suffers from noise and sampling issues, which are reflected in the accuracy of the resulting coevolutionary models (Kleeorin et al. 2023; Cocco et al. 2024). Note that, while in contact predictions only highly coevolving pairs are considered, in studying the mutational landscape, in principle, all possible couplings are taken into account, thus amplifying the effect of potential noisy inference. We thus performed several checks to verify the aforementioned behaviors on our dataset.

We assessed whether epistatic models are more sensitive to MSA depth than independent-site methods. As shown in Table 2, ArDCA appears to be much more sensitive to MSA depth than other models, with correlations as low as 0.25 for proteins with the shallow MSA ($D_{10}$) and as high as 0.53 for proteins with the largest MSA ($D_{10,000}$). However, other epistatic and independent-site models show a similar sensitivity to MSA depth, and this feature alone cannot account for the fact that the more complex epistatic models do not outperform the simpler independent-site models. To further support these observations, we performed an MSA subsampling analysis, which confirmed ArDCA’s high sensitivity to MSA depth compared with other methods (see supplementary section S6.1, Supplementary Material online).

MSA curation is also important in epistatic model inference. For example, removing MSA columns with a high gap frequency can improve the performance of the inferred epistatic model, as it is expected to reduce the noise in the coupling parameters, while having no effect on independent-site models. To show this, we performed MSA trimming by removing columns with gap frequency >0.2 and found an improvement of the epistatic model performances (results reported in supplementary section S6.2, Supplementary Material online). This indicates that epistatic couplings are more sensitive than independent-site models to noise in MSAs.

When inferring an epistatic model, we also have to address the well-known issue of undersampling (Weigt et al. 2009; Morcos et al. 2011; Kleeorin et al. 2023), as the number of parameters usually largely exceeds the number of sequences in the input MSA. Statistical regularization is commonly employed to prevent overfitting by adding penalty terms. These terms play an important role, as is well known in the DCA literature (Hopf et al. 2017; Trinquier et al. 2021; Kleeorin et al. 2023). It has been shown that with different regularization strengths, the inference focuses on different features, shifting from local interactions to larger-scale functionally critical regions (Kleeorin et al. 2023). In supplementary section S6.3, Supplementary Material online, we show how the choice of the regularization parameters can enhance the ability of epistatic model to predict thermodynamic stability. Interestingly, we also found that different regularization strengths have opposite effects on predicting mutations in the core and on the surface of proteins. Weak regularization tends to improve core residue predictions, while strong regularization is optimal for surface residues. There is currently no clearly defined strategy for selecting the best regularization parameters to improve epistatic model performance.

**Fig. 5.**
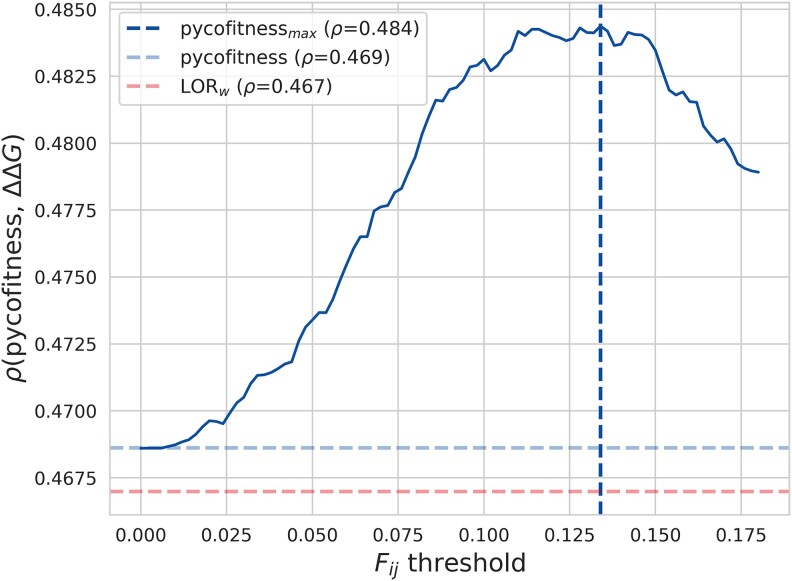
Average per-protein Spearman correlation coefficient *ρ* between the experimental ΔΔG values and the Frobenius norm-modified pycofitness score as a function of the Frobenius norm threshold *t* on dataset D. Horizontal lines represent baseline comparisons: ${\text{LOR}}_{w}$ score in red and default pycofitness score in blue. The vertical line represents the optimal Frobenius norm threshold *t*.

As all our analyses suggest that the epistatic models suffer from noisy couplings to the extent that we do not observe any significant advantage over independent-site methods, we further investigated this point by developing a new epistatic model that includes only a subset of couplings. A similar approach was employed in Cocco et al. (2024), where a supervised selection strategy was used to retain only couplings that enhance performance. In our model, we used an unsupervised approach that retains only coupling terms corresponding to highly coevolving residue pairs (see supplementary section S6.4, Supplementary Material online). Specifically, we measured the coupling strength between two residue positions *i* and *j* by their Frobenius norm $F_{ij}$, and only considered epistatic couplings between positions pairs whose Frobenius norm exceeds a given threshold. We found that this approach significantly improves model performance, as shown in Fig. 5, leading to better performances than independent-site models. More details can be found in supplementary section S6.4, Supplementary Material online.

In summary, our results indicate that current full epistatic models do not improve performance in predicting the impact of mutations on thermodynamic stability compared with independent-site methods due to noisy couplings. However, note that, when predicting other functional properties, such as fitness, this seems to be less the case, as shown in previous analyses (Figliuzzi et al. 2016), which suggests that epistatic models may be better at capturing functional effects than stability.

### RSA and Protein Stability

It is well known that RSA plays an essential role in shaping protein stability, as substitutions in the core, where residues are more constrained by multiple interactions with surrounding residues, tend to be more destabilizing than those at the surface. As a result, many state-of-the-art stability change predictors (such as Dehouck et al. 2011; Laimer et al. 2015; Chen et al. 2020b; Zhou et al. 2023) directly or indirectly use RSA as a feature in their model. The large-scale and systematic nature of the deep mutational scanning dataset D enables a more comprehensive exploration and quantification of this relationship. Furthermore, the analysis in this section offers an explanation of how combining RSA with evolutionary metrics enhances ΔΔG predictions, highlighting that this effect is particularly pronounced for stability compared with other mutational properties like binding or activity.

**Fig. 6.**
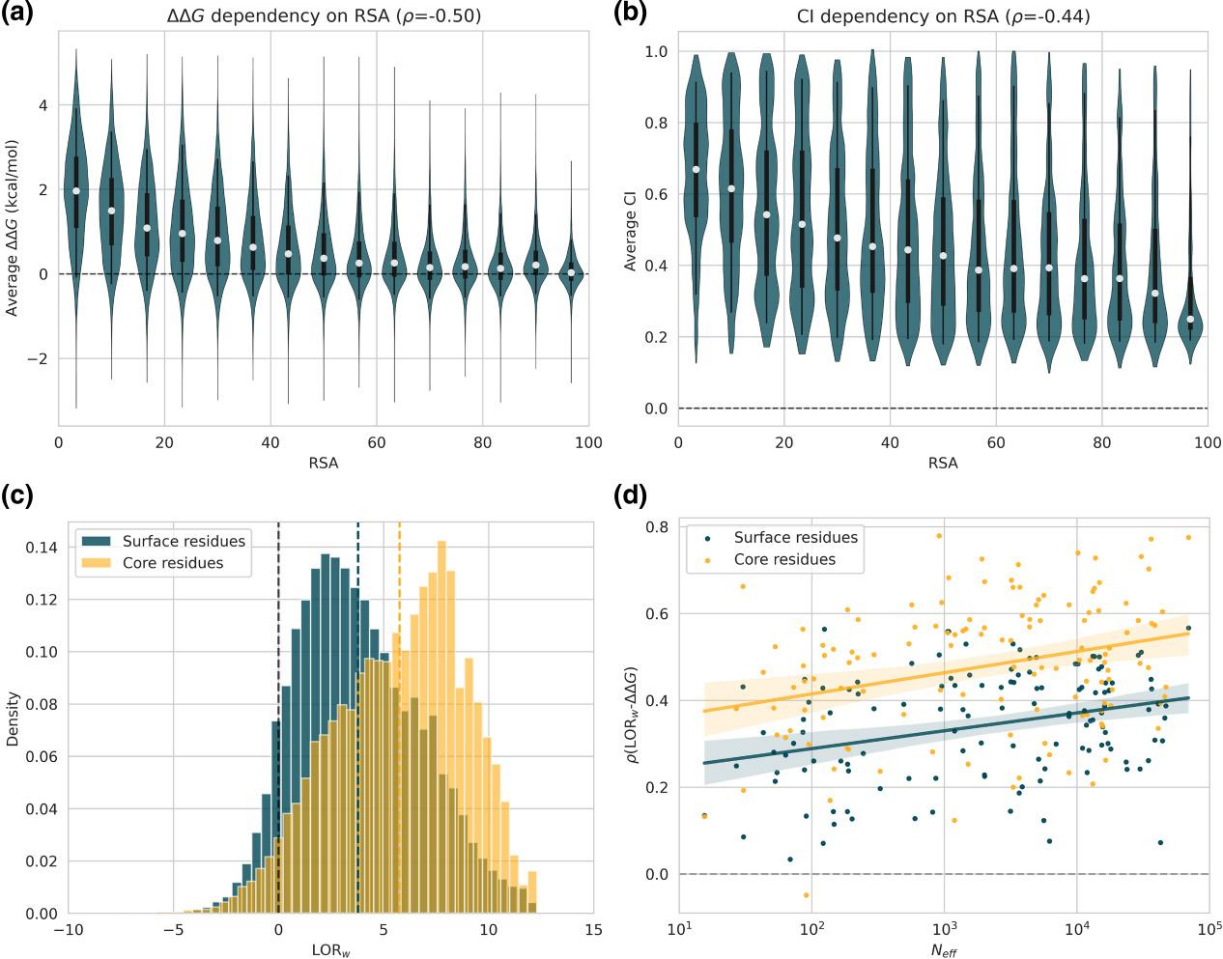
Distributions of a) experimental ΔΔG values in dataset D and b) corresponding CI values as a function of the RSA of the wild-type residues. White dots represent the median, boxes (thick black lines) represent the inter-quartile range from quartile 1 to quartile 3, and whiskers (thin black lines) represent the inter-percentile range from percentile 5 to percentile 95. c) Distribution of ${\text{LOR}}_{w}$ for mutations located in the core (in orange) and at the surface (in blue). d) Per-protein Spearman correlation coefficients *ρ* between ${\text{LOR}}_{w}$ and experimental ΔΔG values for mutations located in the core (in orange) and at the surface (in blue) as a function of $N_{\mathrm{eff}}$ (in $\log_{10}$ scale) in the corresponding MSA.

We first emphasize the notable anticorrelation of −0.50 between RSA and ΔΔG. As shown in Fig. 6a, mutations in low RSA regions display a wide distribution with high destabilizing median values, while mutations in high RSA regions display a narrow distribution with a median only slightly above zero kcal/mol.

The RSA dependence is particularly strong for the effect of variants on stability. This can be seen by examining the RSA dependence of single-site mutation scores from ProteinGym (Notin et al. 2024), a large-scale standard dataset on the effect of protein variants on fitness, which collects many multilabel deep mutational scanning experiments. RSA shows an average per-protein Spearman correlation of −0.50 on mutagenesis data from stability-based assays (essentially composed of values from Tsuboyama et al. (2023) similar to our dataset D). In contrast, data from other type of assays exhibit still remarkable but much weaker average correlations equal to −0.27, −0.28, −0.32, and −0.33 for binding-, organismal fitness-, expression-, and activity-based assays, respectively.

We note that the observed anticorrelation between RSA and ΔΔG may be overestimated on the D dataset because it contains only small monomeric protein domains. In general, larger proteins have a larger fraction of their residues with near-zero RSA values. To test this, we analyzed the 707 mutations from the L dataset, inserted in proteins ranging in length from 108 to 452 residues. We found that the average per-protein Spearman correlation between RSA and ΔΔG is only slightly smaller, with a value of −0.42 (see supplementary section S2, Supplementary Material online for details).

Values of RSA are also strongly related to evolution, as residues at the surface tend to evolve much faster than those in the core (Franzosa and Xia 2012; Yeh et al. 2014; Schwersensky et al. 2020). This is confirmed by the anticorrelation of −0.44 between RSA and the conservation index CI (Fig. 6b).

Unconserved average values for high RSA regions are explained by the fact that such residues are mostly located in variable loop regions or at the C- and N-terminus of the protein, where the evolutionary pressure is low compared with the higher evolutionary pressure that is exerted on core residues. From the perspective of the mutational evolutionary score ${\text{LOR}}_{w}$, we found its average value to be notably higher for core residues (RSA<20%) than for surface residues (RSA≤20%) with averages of 5.75 and 3.78, respectively, as shown in Fig. 6c. Moreover, ${\text{LOR}}_{w}$ displays a stronger predictive power on ΔΔG for core residues than for surface residues (ρ=0.48 and ρ=0.34 respectively, see Fig. 6d). However, other factors may influence this result. First, surface residues are submitted to strong evolutionary pressures from other protein properties such as the optimization of the binding to other proteins, ligands or nucleic acids. Second, since surface residues display smaller stability variations upon mutations, these effects are more subtle and thus more difficult to predict.

### Combining RSA and Evolution for ΔΔG Prediction

As shown in the previous subsection, RSA is strongly related to both protein stability and conservation. The higher evolutionary pressure for stability in the core of proteins makes RSA a promising indicator to modulate signals extracted from MSAs. We therefore used the RSA as defined in Eq. 11 in order to improve the predictive power of the evolutionary scores for ΔΔG prediction.

In addition, the RSA factor helps to mitigate predictions in MSA regions with a high gap ratio. Indeed, protein loop regions and the C- and N-termini tend to be poorly conserved, leading to variable MSAs with many gaps. While mutations in these flexible and exposed regions are often associated with neutral ΔΔG values, they tend to be incorrectly classified as destabilizing by evolutionary models due to the lack of specific sequence information in these regions. Inclusion of the RSA factor helps to lower the predicted scores, which then better describe the tendency of these mutations to be neutral.

**Fig. 7.**
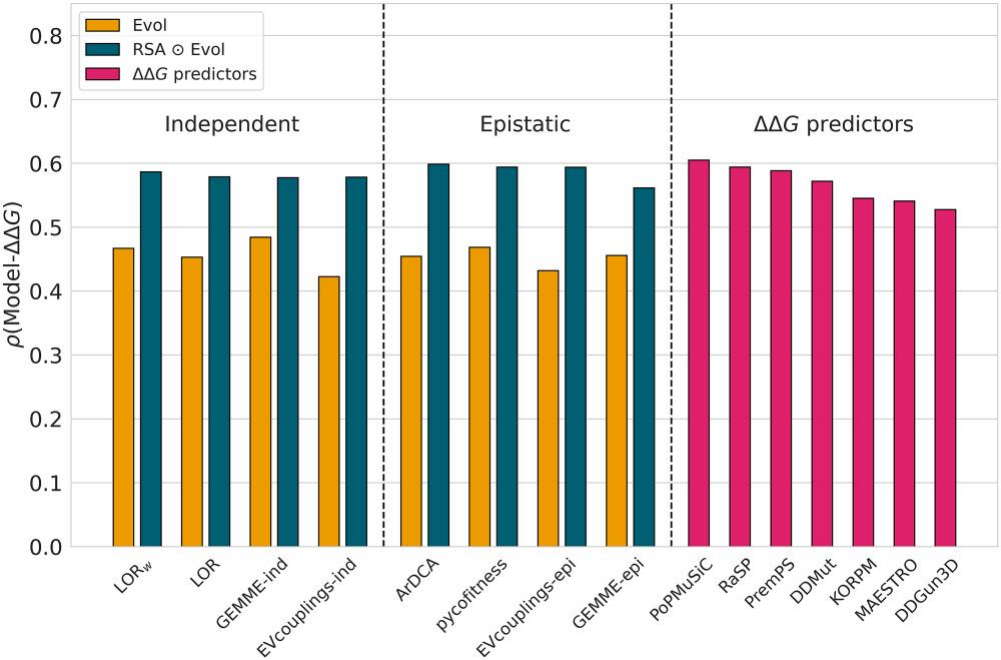
Average per-protein Spearman correlation coefficients *ρ* between experimental ΔΔG values and evolutionary scores (in yellow), evolutionary scores combined with RSA (in dark blue) and predicted ΔΔG values (in pink), on the variant dataset D.

The performance of all evolutionary methods combined with RSA to predict protein stability changes on the dataset D is presented in Fig. 7; the correlation values are provided in supplementary section S7, Supplementary Material online, and the *P*-values for pairwise correlation comparisons are given in supplementary section S3, Supplementary Material online. Additionally, we compared them with seven state-of-the-art ΔΔG prediction models (PoPMuSiC Dehouck et al. 2011, KORPM Hernández et al. 2023, MAESTRO Laimer et al. 2015, 2016, RaSP Blaabjerg et al. 2023, PremPS Chen et al. 2020b, DDMut Zhou et al. 2023, and DDGun3D Montanucci et al. 2019). The first four predictors are purely structure-based; they rely solely on biophysical and statistical features and identify the parameters of their models based on experimental ΔΔG datasets. In contrast, the last three methods include evolutionary features in addition to structure-based features. PremPS extracts them from MSAs, whereas DDGun3D and DDMut use general evolutionary metrics such as mutation matrices and sequence-based statistical potentials. Notably, DDGun3D is almost unsupervised as it only uses experimental ΔΔG datasets to set the weight coefficient between structural and evolutionary features. Also, only RaSP and DDMut use complex deep learning techniques. It is interesting to observe the emergence of transformer-based protein large language models (pLLMs) such as PROSTATA (Umerenkov et al. 2023) and Stability Oracle (Diaz et al. 2024), which show promising performances. However, their learning sets partially rely on the deep mutational scanning dataset (Tsuboyama et al. 2023) from which we constructed our test dataset D, and therefore these methods cannot be used for a fair comparison in our analysis.

Modulation by RSA significantly improves the performance of all evolutionary models, increasing the correlations from *ρ* in the range [0.42,0.48] up to [0.56,0.60]. Surprisingly, these scores are comparable or in some cases even better than those of the supervised ΔΔG prediction models. For example, the best evolutionary score of 0.60 achieved by RSA⊙ArDCA outperforms nearly all ΔΔG predictors and is statistically comparable to the three top-performing predictors (PoPMuSiC, RaSP, and PremPS with no statistically significant difference in *P*-values). As shown in supplementary section S2, Supplementary Material online, this high correlation is also confirmed for mutations in larger proteins from the L dataset, with an average per-protein Spearman correlation of 0.58 for $\text{RSA}⊙{\text{LOR}}_{w}$.

Overall, independent-site and epistatic models achieve comparable performance when modulated by RSA. However, we note that in this case, three epistatic methods (ArDCA, pycofitness, and EVcouplings-epi) outperform all independent-site methods, with relatively small but statistically significant differences (especially on proteins with large $N_{\mathrm{eff}}$). In terms of MSA depth, the RSA-modulated models, as expected, behave similarly to the evolutionary models alone, with performance improving as MSA depth increases (see supplementary section S7, Supplementary Material online). For shallow MSAs, their performance is only slightly better than RSA alone, indicating the weak quality of the extracted (co)evolutionary signals. In contrast, for deeper MSAs, the performance is significantly higher.

In conclusion, in the era of deep learning and large language models, it is impressive to see that simple unsupervised models including only amino acid frequencies and their localization in the structure achieve state-of-the-art performance.

## Conclusion

Evolution and protein stability are closely related. Indeed, protein stability is known to be one of the major factors driving evolution, and on the other hand, evolution imposes constraints on protein stability. In this paper, we took advantage of the huge mutational stability dataset published in Tsuboyama et al. (2023) to analyze how evolutionary information can be leveraged to predict the effects of mutations on protein stability. The main findings are summarized below.

- The quality of the used MSA, particularly its depth, plays a fundamental role in the ability of evolution-based methods to extract information about protein stability. Although this result was expected, the large-scale nature of the dataset used allowed us to quantify these phenomena in a more robust way. We showed that proteins with deeper MSAs tend to be largely better predicted than proteins with shallow MSAs. However, adding sequence data to MSA does not necessarily lead to better scores, as already highlighted in Abakarova et al. (2023).
- Among the various evolutionary models tested, we found that independent-site models perform as well as coevolutionary models, challenging the common belief that the latter should be inherently superior due to their ability to account for interactions between residues. Although coevolutionary models achieve very good performance in predicting 3D contacts between residues, they do not appear to offer an advantage over independent-site models in predicting changes in protein stability.
- Epistatic models have the potential to improve the performance of independent-site models in predicting protein stability. However, we highlight several key factors that hinder this improvement. First, the inference of epistatic models, being inherently complex, often suffers from convergence and undersampling problems. Second, the intrinsic noise in the input MSA (for instance, in columns with a high fraction of gaps) can lead to noisy DCA coupling estimates. Various strategies, including MSA curation, parameter selection, and the choice of a specific subset of couplings, can be employed to improve the performance of epistatic models.
- We further observed that RSA is a key feature that strongly anticorrelates with changes in protein stability. Notably, the simple unsupervised combination of RSA with the analyzed evolutionary features achieves performances comparable to that of state-of-the-art supervised ΔΔG predictors.

In summary, we have demonstrated that evolutionary data can be effectively used in an unsupervised way to predict the impact of mutations on protein stability. Since the performance achieved by modulating evolutionary signals with RSA is comparable to that of complex supervised ΔΔG predictors, an immediate perspective is to combine these models with the aim of further improving ΔΔG prediction. Moreover, as the performance of independent-site vs. epistatic models in predicting the effect of protein mutations is still a topic of debate, our results contribute to clarifying key issues in understanding protein mutational landscapes, shedding light on why epistatic models do not outperform independent-site approaches.

## Supplementary Material

msae267_Supplementary_Data

## Data Availability

All the data generated and used in this study are available in our GitHub repository: https://github.com/3BioCompBio/EvoStability.

## References

[msae267-B1] Abakarova M, Marquet C, Rera M, Rost B, Laine E. Alignment-based protein mutational landscape prediction: doing more with less. Genome Biol Evol. 2023:15(11):evad201. 10.1093/gbe/evad201. 37936309 PMC10653582

[msae267-B2] Alexander H, Hu SK, Krinos AI, Pachiadaki M, Tully BJ, Neely CJ, Reiter T. Eukaryotic genomes from a global metagenomic data set illuminate trophic modes and biogeography of ocean plankton. mBio. 2023:14(6):e01676–23. 10.1128/mbio.01676-23. 37947402 PMC10746220

[msae267-B3] Arnold FH . Directed evolution: bringing new chemistry to life. Angew Chem Int Ed. 2018:57(16):4143–4148. 10.1002/anie.v57.16. PMC5901037 29064156

[msae267-B4] Ashenberg O, Gong LI, Bloom JD. Mutational effects on stability are largely conserved during protein evolution. Proc Natl Acad Sci U S A. 2013:110(52):21071–21076. 10.1073/pnas.1314781111. 24324165 PMC3876214

[msae267-B5] Blaabjerg LM, Kassem MM, Good LL, Jonsson N, Cagiada M, Johansson KE, Boomsma W, Stein A, Lindorff-Larsen K. Rapid protein stability prediction using deep learning representations. Elife. 2023:12:e82593. 10.7554/eLife.82593. 37184062 PMC10266766

[msae267-B6] Bloom JD, Labthavikul ST, Otey CR, Arnold FH. Protein stability promotes evolvability. Proc Natl Acad Sci U S A. 2006:103(15):5869–5874. 10.1073/pnas.0510098103. 16581913 PMC1458665

[msae267-B7] Calabrese R, Capriotti E, Fariselli P, Martelli PL, Casadio R. Functional annotations improve the predictive score of human disease-related mutations in proteins. Hum Mutat. 2009:30(8):1237–1244. 10.1002/humu.v30:8. 19514061

[msae267-B8] Camarillo-Guerrero LF, Almeida A, Rangel-Pineros G, Finn RD, Lawley TD. Massive expansion of human gut bacteriophage diversity. Cell. 2021:184(4):1098–1109. 10.1016/j.cell.2021.01.029. 33606979 PMC7895897

[msae267-B9] Chen M, Chen X, Schafer NP, Clementi C, Komives EA, Ferreiro DU, Wolynes PG. Surveying biomolecular frustration at atomic resolution. Nat Commun. 2020a:11(1):5944. 10.1038/s41467-020-19560-9. 33230150 PMC7683549

[msae267-B10] Chen Y, Lu H, Zhang N, Zhu Z, Wang S, Li M. PremPS: predicting the impact of missense mutations on protein stability. PLoS Comput Biol. 2020b:16(12):e1008543. 10.1371/journal.pcbi.1008543. 33378330 PMC7802934

[msae267-B11] Cocco S, Feinauer C, Figliuzzi M, Monasson R, Weigt M. Inverse statistical physics of protein sequences: a key issues review. Rep Prog Phys. 2018:81(3):032601. 10.1088/1361-6633/aa9965. 29120346

[msae267-B12] Cocco S, Posani L, Monasson R. Functional effects of mutations in proteins can be predicted and interpreted by guided selection of sequence covariation information. Proc Natl Acad Sci U S A. 2024:121(26):e2312335121. 10.1073/pnas.2312335121. 38889151 PMC11214004

[msae267-B13] Coluzza I . Computational protein design: a review. J Phys Condens Matter. 2017:29(14):143001. 10.1088/1361-648X/aa5c76. 28140371

[msae267-B14] Dalkas GA, Teheux F, Kwasigroch JM, Rooman M. Cation–*π*, amino–*π*, *π–π*, and H-bond interactions stabilize antigen–antibody interfaces. Proteins Struct Funct Bioinformatics. 2014:82(9):1734–1746. 10.1002/prot.v82.9. 24488795

[msae267-B15] Dasmeh P, Serohijos AW. Estimating the contribution of folding stability to nonspecific epistasis in protein evolution. Proteins Struct Funct Bioinformatics. 2018:86(12):1242–1250. 10.1002/prot.v86.12. 30039542

[msae267-B16] Dehouck Y, Kwasigroch JM, Gilis D, Rooman M. PoPMuSiC 2.1: a web server for the estimation of protein stability changes upon mutation and sequence optimality. BMC Bioinformatics. 2011:12(1):1–12. 10.1186/1471-2105-12-151. 21569468 PMC3113940

[msae267-B17] Diaz DJ, Gong C, Ouyang-Zhang J, Loy JM, Wells J, Yang D, Ellington AD, Dimakis AG, Klivans AR. Stability oracle: a structure-based graph-transformer framework for identifying stabilizing mutations. Nat Commun. 2024:15(1):6170. 10.1038/s41467-024-49780-2. 39043654 PMC11266546

[msae267-B18] Ekeberg M, Lövkvist C, Lan Y, Weigt M, Aurell E. Improved contact prediction in proteins: using pseudolikelihoods to infer potts models. Phys Rev E Stat Nonlin Soft Mat Phys. 2013:87(1):1–16. 10.1103/PhysRevE.87.012707. 23410359

[msae267-B19] Fariselli P, Martelli PL, Savojardo C, Casadio R. Inps: predicting the impact of non-synonymous variations on protein stability from sequence. Bioinformatics. 2015:31(17):2816–2821. 10.1093/bioinformatics/btv291. 25957347

[msae267-B20] Figliuzzi M, Jacquier H, Schug A, Tenaillon O, Weigt M. Coevolutionary landscape inference and the context-dependence of mutations in beta-lactamase TEM-1. Mol Biol Evol. 2016:33(1):268–280. 10.1093/molbev/msv211. 26446903 PMC4693977

[msae267-B21] Finn RD, Clements J, Eddy SR. HMMER web server: interactive sequence similarity searching. Nucleic Acids Res. 2011:39(suppl_2):W29–W37. 10.1093/nar/gkr367. 21593126 PMC3125773

[msae267-B22] Franzosa EA, Xia Y. Independent effects of protein core size and expression on residue-level structure-evolution relationships. PLoS One. 2012:7(10):e46602. 10.1371/journal.pone.0046602. 23056364 PMC3463513

[msae267-B23] Fu L, Niu B, Zhu Z, Wu S, Li W. CD-HIT: accelerated for clustering the next-generation sequencing data. Bioinformatics. 2012:28(23):3150–3152. 10.1093/bioinformatics/bts565. 23060610 PMC3516142

[msae267-B24] Gerasimavicius L, Liu X, Marsh JA. Identification of pathogenic missense mutations using protein stability predictors. Sci Rep. 2020:10(1):1–10. 10.1038/s41598-020-72404-w. 32958805 PMC7506547

[msae267-B25] Goldstein RA, Pollock DD. Sequence entropy of folding and the absolute rate of amino acid substitutions. Nat Ecol Evol. 2017:1(12):1923–1930. 10.1038/s41559-017-0338-9. 29062121 PMC5701738

[msae267-B26] Hernández IM, Dehouck Y, Bastolla U, López-Blanco JR, Chacón P. Predicting protein stability changes upon mutation using a simple orientational potential. Bioinformatics. 2023:39(1):btad011. 10.1093/bioinformatics/btad011. 36629451 PMC9850275

[msae267-B27] Høie MH, Cagiada M, Frederiksen AHB, Stein A, Lindorff-Larsen K. Predicting and interpreting large-scale mutagenesis data using analyses of protein stability and conservation. Cell Rep. 2022:38(2):110207. 10.1016/j.celrep.2021.110207. 35021073

[msae267-B28] Hopf TA, Green AG, Schubert B, Mersmann S, Schärfe CP, Ingraham JB, Toth-Petroczy A, Brock K, Riesselman AJ, Palmedo P, et al. The EVcouplings python framework for coevolutionary sequence analysis. Bioinformatics. 2019:35(9):1582–1584. 10.1093/bioinformatics/bty862. 30304492 PMC6499242

[msae267-B29] Hopf TA, Ingraham JB, Poelwijk FJ, Schärfe CPI, Springer M, Sander C, Marks DS. Mutation effects predicted from sequence co-variation. Nat Biotechnol. 2017:35(2):128–135. 10.1038/nbt.3769. 28092658 PMC5383098

[msae267-B30] Hou Q, Pucci F, Pan F, Xue F, Rooman M, Feng Q. Using metagenomic data to boost protein structure prediction and discovery. Comput Struct Biotechnol J. 2022:20:434–442. 10.1016/j.csbj.2021.12.030. 35070166 PMC8760478

[msae267-B31] Hou Q, Rooman M, Pucci F. Enzyme stability-activity trade-off: new insights from protein stability weaknesses and evolutionary conservation. J Chem Theory Comput. 2023:35(2):128–135. 10.1021/acs.jctc.3c00036. 37276063

[msae267-B32] Iqbal S, Pérez-Palma E, Jespersen JB, May P, Hoksza D, Heyne HO, Ahmed SS, Rifat ZT, Rahman MS, Lage K, et al. Comprehensive characterization of amino acid positions in protein structures reveals molecular effect of missense variants. Proc Natl Acad Sci U S A. 2020:117(45):28201–28211. 10.1073/pnas.2002660117. 33106425 PMC7668189

[msae267-B33] Jumper J, Evans R, Pritzel A, Green T, Figurnov M, Ronneberger O, Tunyasuvunakool K, Bates R, Žídek A, Potapenko A, et al. Highly accurate protein structure prediction with AlphaFold. Nature. 2021:596(7873):583–589. 10.1038/s41586-021-03819-2. 34265844 PMC8371605

[msae267-B34] Kabsch W, Sander C. Dictionary of protein secondary structure: pattern recognition of hydrogen-bonded and geometrical features. Biopolymers Orig Res Biomolecules. 1983:22(12):2577–2637. 10.1002/bip.v22:12. 6667333

[msae267-B35] Kleeorin Y, Russ WP, Rivoire O, Ranganathan R. Undersampling and the inference of coevolution in proteins. Cell Syst. 2023:14(3):210–219. 10.1016/j.cels.2022.12.013. 36693377 PMC10911952

[msae267-B36] Korendovych IV . Rational and semirational protein design. Protein Eng Methods Protoc. 2018:1685:15–23. 10.1007/978-1-4939-7366-8. PMC5912912 29086301

[msae267-B37] Kurahashi R, Sano S, Takano K. Protein evolution is potentially governed by protein stability: directed evolution of an esterase from the hyperthermophilic archaeon Sulfolobus tokodaii. J Mol Evol. 2018:86(5):283–292. 10.1007/s00239-018-9843-y. 29679096

[msae267-B38] Laimer J, Hiebl-Flach J, Lengauer D, Lackner P. MAESTROweb: a web server for structure-based protein stability prediction. Bioinformatics. 2016:32(9):1414–1416. 10.1093/bioinformatics/btv769. 26743508

[msae267-B39] Laimer J, Hofer H, Fritz M, Wegenkittl S, Lackner P. MAESTRO-multi agent stability prediction upon point mutations. BMC Bioinformatics. 2015:16(1):1–13. 10.1186/s12859-015-0548-6. 25885774 PMC4403899

[msae267-B40] Laine E, Karami Y, Carbone A. Gemme: a simple and fast global epistatic model predicting mutational effects. Mol Biol Evol. 2019:36(11):2604–2619. 10.1093/molbev/msz179. 31406981 PMC6805226

[msae267-B41] Levy Karin E, Mirdita M, Söding J. MetaEuk-sensitive, high-throughput gene discovery, and annotation for large-scale eukaryotic metagenomics. Microbiome. 2020:8(1):1–15. 10.1186/s40168-020-00808-x. 32245390 PMC7126354

[msae267-B42] Ma B, Lu C, Wang Y, Yu J, Zhao K, Xue R, Ren H, Lv X, Pan R, Zhang J, et al. A genomic catalogue of soil microbiomes boosts mining of biodiversity and genetic resources. Nat Commun. 2023:14(1):7318. 10.1038/s41467-023-43000-z. 37951952 PMC10640626

[msae267-B43] Malakauskas SM, Mayo SL. Design, structure and stability of a hyperthermophilic protein variant. Nat Struct Biol. 1998:5(6):470–475. 10.1038/nsb0698-470. 9628485

[msae267-B44] Mirdita M, Schütze K, Moriwaki Y, Heo L, Ovchinnikov S, Steinegger M. Colabfold: making protein folding accessible to all. Nat Methods. 2022:19(6):679–682. 10.1038/s41592-022-01488-1. 35637307 PMC9184281

[msae267-B45] Mitchell AL, Almeida A, Beracochea M, Boland M, Burgin J, Cochrane G, Crusoe MR, Kale V, Potter SC, Richardson LJ, et al. MGnify: the microbiome analysis resource in 2020. Nucleic Acids Res. 2020:48(D1):D570–D578. 10.1093/nar/gkz1035. 31696235 PMC7145632

[msae267-B46] Montanucci L, Capriotti E, Frank Y, Ben-Tal N, Fariselli P. DDGun: an untrained method for the prediction of protein stability changes upon single and multiple point variations. BMC Bioinformatics. 2019:20(S14):1–10. 10.1186/s12859-019-2923-1. 31266447 PMC6606456

[msae267-B47] Morcos F, Pagnani A, Lunt B, Bertolino A, Marks DS, Sander C, Zecchina R, Onuchic JN, Hwa T, Weigt M. Direct-coupling analysis of residue coevolution captures native contacts across many protein families. Proc Natl Acad Sci U S A. 2011:108(49):E1293–E1301. 10.1073/pnas.1111471108. 22106262 PMC3241805

[msae267-B48] Nayfach S, Páez-Espino D, Call L, Low SJ, Sberro H, Ivanova NN, Proal AD, Fischbach MA, Bhatt AS, Hugenholtz P, et al. Metagenomic compendium of 189,680 DNA viruses from the human gut microbiome. Nat Microbiol. 2021:6(7):960–970. 10.1038/s41564-021-00928-6. 34168315 PMC8241571

[msae267-B49] Notin P, Kollasch A, Ritter D, Van Niekerk L, Paul S, Spinner H, Rollins N, Shaw A, Orenbuch R, Weitzman R, et al. ProteinGym: large-scale benchmarks for protein fitness prediction and design. Adv Neural Inf Process Syst. 2024:36. https://proceedings.neurips.cc/paper_files/paper/2023/hash/cac723e5ff29f65e3fcbb0739ae91bee-Abstract-Datasets_and_Benchmarks.html.

[msae267-B50] Olson CA, Wu NC, Sun R. A comprehensive biophysical description of pairwise epistasis throughout an entire protein domain. Curr Biol. 2014:24(22):2643–2651. 10.1016/j.cub.2014.09.072. 25455030 PMC4254498

[msae267-B51] Ota N, Kurahashi R, Sano S, Takano K. The direction of protein evolution is destined by the stability. Biochimie. 2018:150:100–109. 10.1016/j.biochi.2018.05.006. 29775634

[msae267-B52] Ovchinnikov S, Park H, Varghese N, Huang P-S, Pavlopoulos GA, Kim DE, Kamisetty H, Kyrpides NC, Baker D. Protein structure determination using metagenome sequence data. Science. 2017:355(6322):294–298. 10.1126/science.aah4043. 28104891 PMC5493203

[msae267-B53] Packer MS, Liu DR. Methods for the directed evolution of proteins. Nat Rev Genet. 2015:16(7):379–394. 10.1038/nrg3927. 26055155

[msae267-B54] Park Y, Metzger BP, Thornton JW. Epistatic drift causes gradual decay of predictability in protein evolution. Science. 2022:376(6595):823–830. 10.1126/science.abn6895. 35587978 PMC9429997

[msae267-B55] Petti S, Bhattacharya N, Rao R, Dauparas J, Thomas N, Zhou J, Rush AM, Koo P, Ovchinnikov S. End-to-end learning of multiple sequence alignments with differentiable Smith–Waterman. Bioinformatics. 2023:39(1):btac724. 10.1093/bioinformatics/btac724. 36355460 PMC9805565

[msae267-B56] Pires DE, Ascher DB, Blundell TL. mCSM: predicting the effects of mutations in proteins using graph-based signatures. Bioinformatics. 2014:30(3):335–342. 10.1093/bioinformatics/btt691. 24281696 PMC3904523

[msae267-B57] Pollock DD, Goldstein RA. Strong evidence for protein epistasis, weak evidence against it. Proc Natl Acad Sci U S A. 2014:111(15):E1450–E1450. 10.1073/pnas.1401112111. 24706894 PMC3992644

[msae267-B58] Pollock DD, Thiltgen G, Goldstein RA. Amino acid coevolution induces an evolutionary Stokes shift. Proc Natl Acad Sci U S A. 2012:109(21):E1352–E1359. 10.1073/pnas.1120084109. 22547823 PMC3361410

[msae267-B59] Pucci F, Schwersensky M, Rooman M. Artificial intelligence challenges for predicting the impact of mutations on protein stability. Curr Opin Struct Biol. 2022:72:161–168. 10.1016/j.sbi.2021.11.001. 34922207

[msae267-B60] Pucci F, Zerihun MB, Peter EK, Schug A. Evaluating DCA-based method performances for rna contact prediction by a well-curated data set. RNA. 2020:26(7):794–802. 10.1261/rna.073809.119. 32276988 PMC7297115

[msae267-B61] Pucci F, Zerihun MB, Rooman M, Schug A. pycofitness—evaluating the fitness landscape of RNA and protein sequences. Bioinformatics. 2024:40(2):btae074. 10.1093/bioinformatics/btae074. 38335928 PMC10881095

[msae267-B62] Raimondi D, Gazzo AM, Rooman M, Lenaerts T, Vranken WF. Multilevel biological characterization of exomic variants at the protein level significantly improves the identification of their deleterious effects. Bioinformatics. 2016:32(12):1797–1804. 10.1093/bioinformatics/btw094. 27153718

[msae267-B63] Rose GD, Geselowitz AR, Lesser GJ, Lee RH, Zehfus MH. Hydrophobicity of amino acid residues in globular proteins. Science. 1985:229(4716):834–838. 10.1126/science.4023714. 4023714

[msae267-B64] Sanavia T, Birolo G, Montanucci L, Turina P, Capriotti E, Fariselli P. Limitations and challenges in protein stability prediction upon genome variations: towards future applications in precision medicine. Comput Struct Biotechnol J. 2020:18:1968–1979. 10.1016/j.csbj.2020.07.011. 32774791 PMC7397395

[msae267-B65] Schwersensky M, Rooman M, Pucci F. Large-scale in silico mutagenesis experiments reveal optimization of genetic code and codon usage for protein mutational robustness. BMC Biol. 2020:18(1):1–17. 10.1186/s12915-020-00870-9. 33081759 PMC7576759

[msae267-B66] Starr TN, Thornton JW. Epistasis in protein evolution. Protein Sci. 2016:25(7):1204–1218. 10.1002/pro.v25.7. 26833806 PMC4918427

[msae267-B67] Steinegger M, Mirdita M, Söding J. Protein-level assembly increases protein sequence recovery from metagenomic samples manyfold. Nat Methods. 2019:16(7):603–606. 10.1038/s41592-019-0437-4. 31235882

[msae267-B68] Suzek BE, Wang Y, Huang H, McGarvey PB, Wu CH, UniProt Consortium. UniRef clusters: a comprehensive and scalable alternative for improving sequence similarity searches. Bioinformatics. 2015:31(6):926–932. 10.1093/bioinformatics/btu739. 25398609 PMC4375400

[msae267-B69] Trinquier J, Uguzzoni G, Pagnani A, Zamponi F, Weigt M. Efficient generative modeling of protein sequences using simple autoregressive models. Nat Commun. 2021:12(1):5800. 10.1038/s41467-021-25756-4. 34608136 PMC8490405

[msae267-B70] Tsuboyama K, Dauparas J, Chen J, Laine E, Behbahani YM, Weinstein JJ, Mangan NM, Ovchinnikov S, Rocklin GJ. Mega-scale experimental analysis of protein folding stability in biology and design. Nature. 2023:620(7973):434–444. 10.1038/s41586-023-06328-6. 37468638 PMC10412457

[msae267-B71] Umerenkov D, Nikolaev F, Shashkova TI, Strashnov PV, Sindeeva M, Shevtsov A, Ivanisenko NV, Kardymon OL. PROSTATA: a framework for protein stability assessment using transformers. Bioinformatics. 2023:39(11):btad671. 10.1093/bioinformatics/btad671. 37935419 PMC10651431

[msae267-B72] Wallner B . Afsample: improving multimer prediction with AlphaFold using massive sampling. Bioinformatics. 2023:39(9):btad573. 10.1093/bioinformatics/btad573. 37713472 PMC10534052

[msae267-B73] Wang Y, Xue P, Cao M, Yu T, Lane ST, Zhao H. Directed evolution: methodologies and applications. Chem Rev. 2021:121(20):12384–12444. 10.1021/acs.chemrev.1c00260. 34297541

[msae267-B74] Watanabe H, Matsumaru H, Ooishi A, Feng Y, Odahara T, Suto K, Honda S. Optimizing pH response of affinity between protein G and IgG Fc: how electrostatic modulations affect protein-protein interactions. J Biol Chem. 2009:284(18):12373–12383. 10.1074/jbc.M809236200. 19269963 PMC2673305

[msae267-B75] Weigt M, White RA, Szurmant H, Hoch JA, Hwa T. Identification of direct residue contacts in protein–protein interaction by message passing. Proc Natl Acad Sci U S A. 2009:106(1):67–72. 10.1073/pnas.0805923106. 19116270 PMC2629192

[msae267-B76] Yeh S-W, Liu J-W, Yu S-H, Shih C-H, Hwang J-K, Echave J. Site-specific structural constraints on protein sequence evolutionary divergence: local packing density versus solvent exposure. Mol Biol Evol. 2014:31(1):135–139. 10.1093/molbev/mst178. 24109601

[msae267-B77] Zeldovich KB, Chen P, Shakhnovich EI. Protein stability imposes limits on organism complexity and speed of molecular evolution. Proc Natl Acad Sci U S A. 2007:104(41):16152–16157. 10.1073/pnas.0705366104. 17913881 PMC2042177

[msae267-B78] Zheng F, Liu Y, Yang Y, Wen Y, Li M. Assessing computational tools for predicting protein stability changes upon missense mutations using a new dataset. Protein Sci. 2024:33(1):e4861. 10.1002/pro.4861. 38084013 PMC10751734

[msae267-B79] Zhou Y, Pan Q, Pires DE, Rodrigues CH, Ascher DB. DDMut: predicting effects of mutations on protein stability using deep learning. Nucleic Acids Res. 2023:51(W1):W122–W128. 10.1093/nar/gkad472. 37283042 PMC10320186

